# Expression analysis and biological characterization of *Babesia* sp. BQ1 (Lintan) (*Babesia motasi*-like) rhoptry-associated protein 1 and its potential use in serodiagnosis via ELISA

**DOI:** 10.1186/s13071-016-1573-7

**Published:** 2016-05-31

**Authors:** Qingli Niu, Zhijie Liu, Jifei Yang, Peifa Yu, Yuping Pan, Bintao Zhai, Jianxun Luo, Emmanuelle Moreau, Guiquan Guan, Hong Yin

**Affiliations:** State Key Laboratory of Veterinary Etiological Biology, Key Laboratory of Veterinary Parasitology of Gansu Province, Lanzhou Veterinary Research Institute, Chinese Academy of Agricultural Science, Xujiaping 1, Lanzhou, Gansu 730046 P. R. China; Jiangsu Co-innovation Center for Prevention and Control of Important Animal Infectious Diseases and Zoonoses, Yangzhou, 225009 P. R. China; INRA, UMR1300 Biology, Epidemiology and Risk Analysis in Animal Health, BP 40706, F-44307 Nantes, France

**Keywords:** *Babesia* sp. BQ1 (Lintan), Rhoptry-associated protein, Biological characterization, Diagnosis

## Abstract

**Background:**

In China, ovine babesiosis is one of the most important tick-borne haemoparasitic diseases of small ruminants. It has a significant economic impact, and several *Babesia motasi*-like isolates have been recently shown to be responsible for ovine babesiosis in this country.

**Methods:**

Full-length and C-terminal-truncated forms of the *rap-1a61-1* gene of *Babesia* sp. BQ1 (Lintan) were cloned into the pET-30a plasmid and subsequently expressed as His-fusion proteins. The resulting recombinant RAP-1a proteins (rRAP-1a61-1 and rRAP-1a61-1/CT) were purified and evaluated as diagnostic antigens using Western blot analysis and ELISA. The native *Babesia* sp. BQ1 (Lintan) RAP-1 protein was recognized using Western blots and IFAT by antibodies that were raised in rabbits against rRAP-1a61-1/CT. The specificity, sensitivity and positive threshold values for rRAP-1a61-1/CT in ELISA were evaluated.

**Results:**

Cross-reactivity was observed between rRAP-1a61-1/CT and positive sera for *Babesia* sp. BQ1 (Lintan), *Babesia* sp. BQ1 (Ningxian) and *Babesia* sp. Tianzhu isolates obtained from infected sheep. At one week post-inoculation, a significant increase was observed in the amount of antibodies produced against RAP-1a, and high levels of antibodies against RAP-1a were observed for 3 months (at 84 days p.i.). A total of 3198 serum samples were collected from small ruminants in 54 different regions in 23 provinces of China. These samples were tested using ELISA based on the rRAP-1a61-1/CT protein. The results indicated that the average positive rate was 36.02 %.

**Conclusions:**

The present study suggests that rRAP-1a61-1/CT might be a potential diagnostic antigen for detecting several isolates of *B. motasi*-like parasites infection.

## Background

Ovine babesiosis is one of the most important tick-borne diseases of small ruminants. This disease has been described in many countries throughout Asia, Europe and Africa [1]. A phylogenetic tree based on the sequence of the 18S rRNA gene showed that *Babesia* species belonging to the following four groups are responsible for babesiosis in small ruminants: *B. ovis*, *B. motasi* which are a European and a Chinese clade (*B. motasi*-like), and the *Babesia* sp. Xinjiang and *B. crassa* [2–7]. In the *B. motasi* Chinese clade (*B. motasi*-like), six *Babesia* isolates (*Babesia* sp. BQ1 (Lintan), *Babesia* sp. BQ1 (Ningxian), *Babesia* sp. Tianzhu, *Babesia* sp. Hebei, *Babesia* sp. Madang and *Babesia* sp. Liaoning) have been recently reported in the Gansu, Hebei and Liaoning provinces of China. These isolates can be classified into one or more sub-groups according to their phylogenetic relationships, which have been reconstructed based on several (18S rRNA, ITS, 28S, *rap-1*) phylogenetic markers [7–12].

Currently, the strategy for controlling babesiosis is based on chemotherapy against either the vector or the parasite. Several compounds are used, but mainly ivermectin is used against ticks and imidocarb against *Babesia*. Several new anti-*Babesia* drugs (Triclosan, Nerolidol, Artesunate and Atovaquone) are also used to control and treat babesiosis [13]. However, this strategy has many drawbacks, such as drug-resistance, the presence of drug metabolites in the milk and meat, and high toxicity to the animals themselves. Optimal treatment methods, including those with biological activity against ticks and vaccines that target either the ticks or the parasites, have been extensively studied. But only live attenuated vaccines are currently used in some countries, and no producing or testing recombinant or subunit vaccines have been available to prevent babesiosis so far [13–16].

Clinical symptoms of babesiosis usually include fever, anemia, and hemoglobinuria and can lead to death in severe cases, which become apparent when merozoites from the *Babesia* invade and replicate within host erythrocytes, resulting in detectable parasitemia [17]. During the process of erythrocytic invasion, some of the proteins, including apical membrane antigen-1 (AMA-1), rhoptry-associated-protein-1 (RAP-1), and spherical body proteins (SBP-1, 2, 3), are secreted by the apical organelles (rhoptries, micronemes and dense granules). Although the functions and implications of these proteins for vaccines designed against *Babesia* infections have been described mainly in *B. bigemina* and *B. bovis*, these data may form a basis for developing a recombinant vaccine against other *Babesia* infections [17].

RAP-1 is an immunogenic protein that is approximately 40–60 kDa in size. It is localized in rhoptries and has been characterized in all *Babesia* species examined so far (*B. bovis*, *B. bigemina*, *B. divergens*, *B. canis*, *B. ovis, B. motasi*-like, *B. orientalis* and *B. gibsoni*) [12, 18–23]. RAP-1 was detected at all asexual growth stages [24] and was expressed during the sporozoite stage by a RAP-1 specific antisera that was obtained from rabbits immunized to neutralize the binding of sporozoites of *B. bovis* to erythrocytes [25]. Moreover, the role of RAP-1 in the erythrocyte invasion process has been studied in vitro during parasite adhesion to erythrocytes, and experiments have been performed to study the inhibitory effect of RAP-1-specific antibodies on growth and invasion [24–26]. Immunization with native and recombinant RAP-1 protein from *B. bovis* and *B. bigemina* also induced immunity and protected animals upon challenge [27–29]. These studies strongly support the notion that RAP-1 plays a functional role in the biology and development of *Babesia* parasites and represents a potential candidate for the development of recombinant vaccines against babesiosis [30–33].

*Rap-1* was characterized and found to be present as multiple gene copies that are arranged in a tandem, head to tail manner in all *Babesia* species. In sheep *Babesia* species, the *rap-1* locus of the *B. motasi*-like gene is similar to the locus of *B. bigemina*: in both species, three different isoforms (*rap-1a*, *rap-1b* and *rap-1c*), 5 identical *rap-1b* copies and a single *rap-1c* located at end of locus have been described [12, 34]. Two *rap-1a* classes named *rap-1a61* and *rap-1a67* have been described, and each class is represented by two close variants (*rap-1a61-1* and *rap-1a61-2*; *rap-1a67-1* and *rap-1a67-2*) in *Babesia* sp. BQ1 (Lintan) [34]. Polymorphisms were limited to three nucleotide positions in the *rap-1a61* copies (*rap-1a61-1*, *-2*, *-3*) and were found in the *rap-1* locus of *Babesia* sp. BQ1 (Lintan) (*rap-1a61-1* and *-2*), *Babesia* sp. BQ1 (Ningxian) and *Babesia* sp. Tianzhu (*rap-1a61-2* and *-3*) [12, 34]. According to a phylogenetic tree that was constructed based on the *rap-1* gene sequences, the *B. motasi*-like group can be separated into two sub-groups: one that consists *Babesia* sp. BQ1 (Lintan and Ningxian) and *Babesia* sp. Tianzhu and another that contains *Babesia* sp. Hebei [12].

The *rap-1a* gene has been found in all *Babesia* species and shows highly conserved protein features (30–45 % identity among species), suggesting that this gene plays an important role in the RBC invasion process [35]. Moreover, RAP-1a, in *Babesia* has been widely used as a vaccine candidate and diagnostic antigen [21, 29, 36, 37]. The expression, biological characterization and immunogenicity of RAP-1a in sheep *Babesia* species have not been extensively studied. In the present study, we cloned and expressed RAP-1a and then detected the presence of antibodies directed against native and recombinant forms of RAP-1a during the course of infection. To evaluate the potential of using recombinant RAP-1a as a diagnostic antigen, we compared the diagnostic potential of full-length and truncated C-terminal recombinant *Babesia* sp. BQ1 (Lintan) RAP-1a proteins using via Western blot analysis. We discuss our results in combination with the outcomes of previous studies.

## Methods

### Parasites

The original isolates of the *B. motasi*-like group, including *Babesia* sp. BQ1 (Lintan), *Babesia* sp. BQ1 (Ningxian), *Babesia* sp. Tianzhu and *Babesia* sp. Hebei, were obtained from infected sheep and then cryopreserved in liquid nitrogen at the Vector and Vector-borne Disease (VVBD) Laboratory of the Lanzhou Veterinary Research Institute (LVRI) (CAAS Lanzhou, China) [3, 4, 6].

### Sera

Positive sera were obtained from five different breeds of sheep (two “Tan mutton” Chinese sheep, including No. 3216 and 2007, and three “Vendean” French sheep, including No. 3309, 3533 and 3446) that were infected with *Babesia* sp. BQ1 (Lintan). Immune-positive sera were obtained from sheep Nos. 08026, 08040 and 08020, which were infected by *Babesia* sp. BQ1 (Ningxian), *Babesia* sp. Tianzhu and *Babesia* sp. Hebei, respectively, and immune-positive sera were obtained from sheep No. 3201, which was infected with *Babesia* sp. Xinjiang, as previously described [38]. These serum samples were used to evaluate cross-infection and antibody kinetics. Positive sera from sheep that were experimentally infected with *Theileria luwenshuni*, *T. uilenbergi* and *Anaplasma ovis* or bovine *B. bovis* and *B. bigemina* were used as the controls to evaluate cross-reactivity.

Fifty serum samples were collected from five sheep (3–12 wpi) that were experimentally infected with *Babesia* sp. BQ1 (Lintan). In total, 141 sera and anticoagulated blood samples were collected from sheep in the Xinjiang, Henan and Gansu provinces of China. Genomic DNA was purified from blood samples and tested using specific PCR primers, and 141 genomic DNA samples were found to be specifically positive for *Babesia* sp. BQ1 (Lintan) in the sequencing analysis (data not shown). The sera samples (*n* = 141) that corresponded to these genomic DNA samples were selected as the positive serum samples. A total of 191 sera were used as positive samples to evaluate the sensitivity of the ELISA, and a mixture of these sera was used as the positive control serum.

Sera were collected from 492 lambs that were purchased from a *Babesia*-free region in Jingtai country (Gansu province of China) from 2010 to 2015. After nested PCR tests were performed, none of the samples tested positive, and a mixture of these serum samples was used as the negative control serum to evaluate the specificity of the ELISA.

**Fig. 1:**
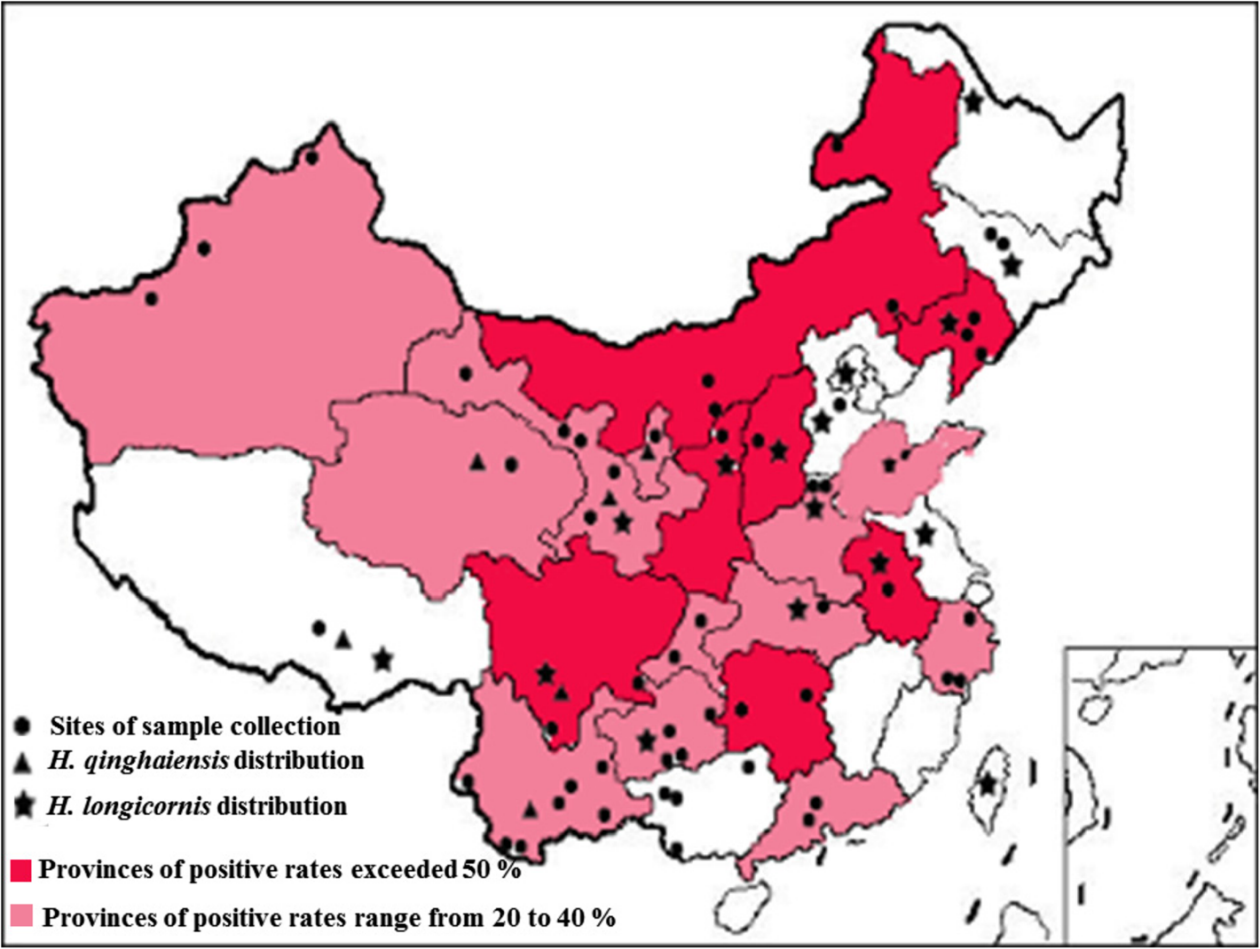
The geographical distribution of the *B. motasi*-like sp. that was identified in this study and the distribution of *Haemaphysalis* spp. in China. The positive rates exceed 50 % indicated with deep red, and the positive rates ranged from 20 to 40 % indicated with light red, less than 10 % infected rates indicated without color

A total of 3198 serum samples were randomly collected from clinically healthy sheep from 54 different locations in 23 Chinese provinces between 2010 and 2015 (Fig. 1). All blood samples were collected in non-anticoagulation tubes and transported to the laboratory while on ice. The serum was then separated and stored at -20 °C until further use.

### Ethical approval

This study was approved by the Animal Ethics Committee of the Lanzhou Veterinary Research Institute at the Chinese Academy of Agricultural Sciences. All animals used in this study were handled in accordance with the Animal Ethics Procedures and Guidelines of the People’s Republic of China.

### Cloning of the full-length and the C-terminal truncated RAP-1a61-1 genes

Genomic DNA was extracted from infected blood samples obtained from sheep infected with *Babesia* sp. BQ1 (Lintan) using a QIAamp DNA Blood Kit (QIAGEN, Maryland, USA) according to the manufacturer’s instructions. These DNA samples were used as the templates for PCR. The oligonucleotide primers that were used to amplify the full-length and C-terminal-truncated *rap-1a61-1* genes were designed based on the DNA sequence of *Babesia* sp. BQ1 (Lintan) RAP-1a61-1 (GenBank accession number KC953701) and modified to include either *Xb*a I and *BamH* I or *Nde* I and *Hind* III (New England BioLabs) enzyme restriction sites (underlined below) at their 5' end. The following primers were used: RAP-1a61-full-F: 5'-CGC TCT AGA -GT ACG CCA TTA CCA CCG TTC-3', RAP-1a61-full-R: 5'-GCC GGA TCC GAG CCA CGA ATC ATC GGA TTC-3', RAP-1a61/CT-F: 5'-CGC CAT ATG ACC ACG GTG ATG CCC GAG-3', and RAP-1a61/CT-R: 5'-GCC AAG CTT GAG CCA CGA ATC ATC GGA-3'. The PCR products were cloned into the pUC57 vector according to the manufacturer’s instructions (Genscript). The inserts were sequenced using vector primers. Representative plasmids were selected and digested using either *Xba* I and *BamH* I or *NdeI* and *HindIII* restriction enzymes and subsequently cloned into digested pET-30a expression vectors (Genscript) according to the manufacturer’s instructions. This resulted in the generation of the recombinant plasmids pET-30a-RAP-1a61-1 and pET-30a-RAP-1a61-1/CT, which contained the full-length gene (1359 bp, except for signal peptide sequence) and the C-terminal-truncated fragment (450 bp), respectively. Both of the cloned RAP-1a proteins were tagged with histidine.

### Expression and purification of the recombinant proteins

To express the recombinant proteins, both pET-30a-RAP-1a61-1 and pET-30a-RAP-1a61-1/CT were transformed into BL21 *E. coli* (DE3 strain) cells. The expression of the His-tagged fusion proteins rRAP-1a61-1 and rRAP-1a61-1/CT was induced in the presence of kanamycin (50 μg/ml) by adding 1 mM IPTG and then culturing the cells at 15 °C for 16 h. The bacterial cultures were harvested and lysed using ultrasonication in binding buffer (50 mM Tris, 6 M Gu-HCl; pH 8.0) containing phenylmethylsulfonyl fluoride (PMSF) and then purified as inclusion bodies from the *E. coli* cells. The target protein was then eluted using urea followed by a series of buffers containing increasing concentrations of imidazole (200 mM, 500 m mM and 2500 m mM). After the proteins were renatured, they were suspended in 1× SDS-PAGE sample loading buffer (62.5 mM Tris-HCl [pH 6.8], 2 % SDS, 5 % β-mercaptoethanol, 10 % glycerol, and 0.02 % bromophenol blue) and boiled for 5 min. The samples were briefly centrifuged prior to being separated using SDS-PAGE. Recombinant protein expression was then analyzed using Western blot analysis.

### Preparation of anti-rRAP-1/CT specific rabbit immune serum

New Zealand Rabbits (2 kg each) were subcutaneously injected with 400 μg of purified rRAP-1a61-1/CT protein that had been emulsified in Freund’s complete adjuvant (FCA, Sigma). In the negative control, the sera were collected from each rabbit prior to the first injections. Booster injections contained the same amount of protein in Freund’s incomplete adjuvant (FIA, Sigma). These injections were administered on days 15, 20 and 28. The sera were collected from the immunized rabbits 15 days after the last injection, purified and stored at -20 °C until further use.

### Western blot analysis

Approximately 20 μg of purified *Babesia* sp. BQ1 (Lintan) recombinant proteins (rRAP-1a61-1 and rRAP-1a61-1/CT) were separated using SDS-PAGE in 18 % polyacrylamide gels. The proteins were transferred to nitrocellulose (NC) membranes with a 0.45 μm pore size (BioRad) at 24 V and 50 W for 35 min. The membrane was then cut into 0.25 cm strips and incubated in a blocking solution [5 % skimmed milk powder in Tris-buffered saline (pH 7.6) with 0.1 % Tween-20 (TBST)] for approximately 3 h at 4 °C on a shaker. After the NC strips had been washed three times, they were incubated for 1 h with each tested serum (diluted at 1:100 in TBST). After the strips were washed three times in TBST, they were incubated for 2 h with monoclonal anti-goat/sheep IgG-alkaline phosphatase conjugates (Sigma, A8062, dilution: 1:5000). The strips were then washed three times with TBST and incubated in a 5-bromo-4-chloro-3-indolyl phosphate/nitro blue tetrazolium (BCIP/NBT) liquid substrate system (B1911-100ML, Sigma) for 15 to 20 min. The approximate molecular weights of the revealed proteins were evaluated by comparing their migration to a standard molecular weight marker (SM0671, 10–170 kDa, PageRuler Prestained Protein Ladder; SM1861, 1.7–40 kDa, Spectra Multicolor Low Range Protein Ladder, Thermo Scientific).

### IFAT and confocal laser microscopic observation

An immunofluorescence analysis was performed using a procedure previously described by Moitra et al. [39] with rabbit antiserum directed against rRAP-1a61-1/CT. The iRBC membrane was stained using a PKH26 red fluorescent cell membrane labeling kit (MINI26, Sigma-Aldrich) according to the manufacturer’s instructions. The iRBCs were fixed in a buffer containing 4 % paraformaldehyde and 10 mM piperazine-N, N-bis (2-ethanesulfonic acid) in PBS (PIPES) at pH 6.4 on 22 mm^2^ poly-L-lysine-coated coverslips for 30 min at room temperature. The coverslips were washed in PBS and then blocked for 1 h in 3 % bovine serum albumin (BSA) in PBS containing 0.25 % Triton X-100. The coverslips were stained overnight at 4 °C with either pre-immune serum or purified anti-rRAP-1a61-1/CT serum (1:50) diluted in blocking buffer without Triton X-100. The coverslips were washed 3 times in PBS, stained for 1 h with FITC-conjugated anti-rabbit IgG (whole molecule) secondary antibodies (Sigma: F0382; 1:80), and then washed 3 times in PBS. The coverslips were stained with 1 μg/ml 4’,6-diamidino-2-phenylindole (DAPI) (Sigma: D8417) for 10 min and then examined for reactivity using confocal microscopy.

### ELISA

The optimum concentration of coating buffer (0.1 M carbonate/bicarbonate, pH 9.6) was determined for rRAP-1a61-1/CT (100 μl at 2 μg/ml). It was then distributed and adsorbed in 96-well flat-bottom microplates at 4 °C overnight. After three washes with PBST (0.1 % Tween-20), the plates were blocked with 200 μl of 2 % gelatin in PBST at 37 °C for 1 h. After the plates were washed, the positive and negative sera (dilution: 1/100) were distributed in duplicate, and the plates were incubated for 1 h at 37 °C. After the plates were washed 3 times, 100 μl of monoclonal anti-goat/sheep IgG-peroxidase (Sigma, A9452, dilution: 1:1000) was added to each well, and the wells were incubated for 1 h at 37 °C. After the plates were washed another 3 times, 100 μl of 1-Step™ Ultra TMB-ELISA (34028–250 ml, Thermo Scientific) was added to each well, and the plates were incubated for 15 to 25 min at room temperature. The reaction was stopped by adding 100 μl of 2 M H_2_SO_4_, and the plates were read at 450 nm using an ELISA automat (Bio-Rad Model 680 microplate reader, USA).

### Statistical analysis

The 95 % confidence intervals (95 % CIs) for the overall prevalence values of *Babesia* sp. BQ1 (Lintan) infection were calculated using IBM SPSS Statistics version 19.0.

## Results

### Cloning and expression of RAP-1a61-1 and C-terminal-truncated RAP-1a61-1

**Fig. 2:**
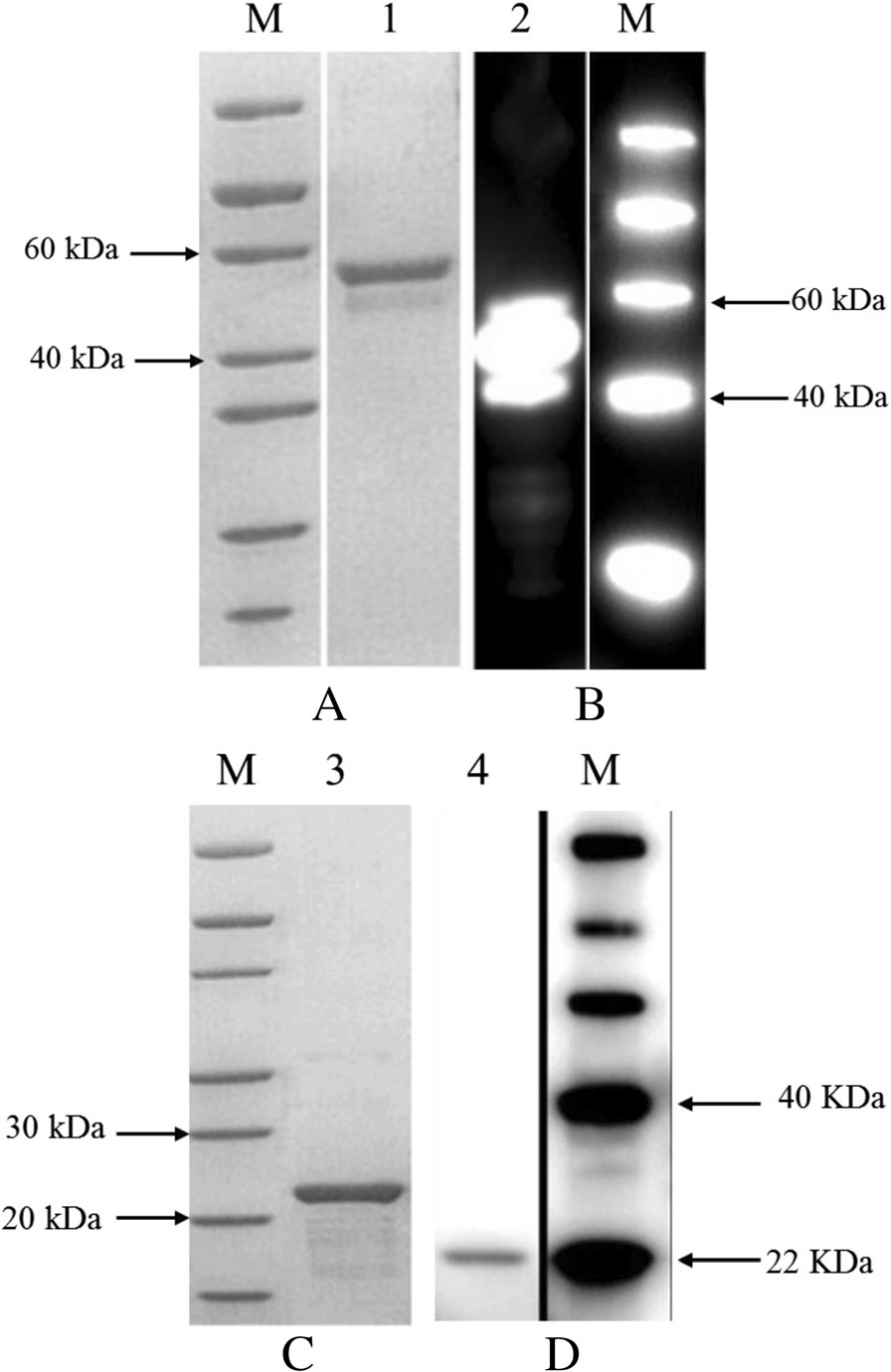
SDS-PAGE and Western blot analysis of the purified rRAP-1a61-1 (Panel **a** and **b**) and C-terminal truncated rRAP-1a61-1/CT (Panel **c** and **d**) proteins with His-tags that were expressed in *E. coli* cultures. M: molecular weight marker. Lanes 1 and 3: SDS-PAGE analysis; Lanes 2 and 4: Western blot analysis using an anti-His antibody

The genes encoding RAP-1a61-1 (aa 22-474) and C-terminal RAP-1a61-1/CT (aa 325-474) (aa sequence corresponding to GenBank accession number AGV15809) were successfully amplified using PCR and inserted into the cloning vector pUC57. The pET-30a-RAP-1a61-1 and pET-30a-RAP-1a61-1/CT recombinant plasmids were identified using enzyme restriction analysis (*Sac* I/*Sal* I and *Mlu* I/*Hind* III, respectively) and confirmed by sequencing using specific primers. The insert sequences were also verified (data not shown). After the expression of each recombinant protein was induced using IPTG, the *E. coli* lysates were analyzed using SDS-PAGE and Western blot analysis of an anti-His serum. The results of SDS-PAGE showed that rRAP-1a61-1 and rRAP-1a61-1/CT were expressed as proteins that were approximately 52 kDa and 20 kDa in size, respectively (Fig. 2).

### Evaluation of the specificity of rRAP-1a61-1 and rRAP-1a61-1/CT using Western blot analysis

**Fig. 3:**
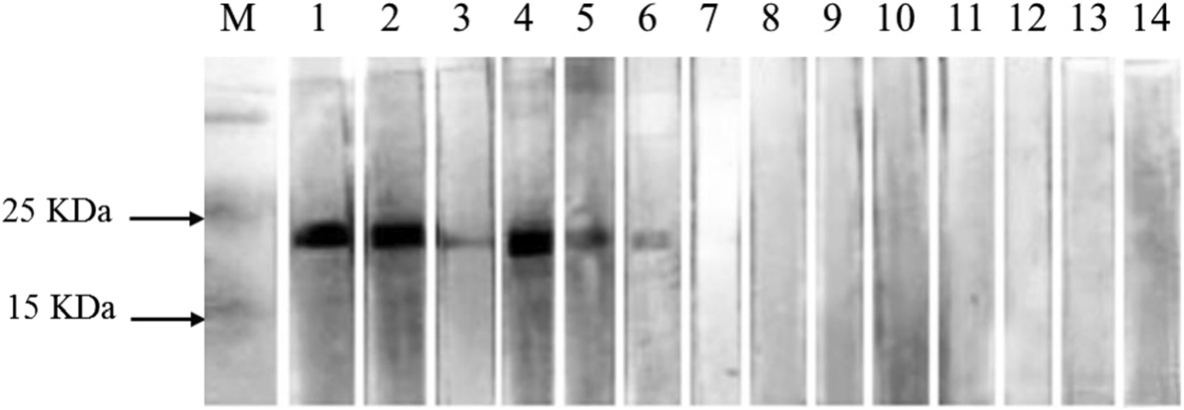
Western blot analysis of the recombinant RAP-1a61-1/CT protein in *Babesia* sp. BQ1 (Lintan). M: molecular weight marker. Lanes 1 and 2: positive sera for *Babesia* sp. BQ1 (Lintan) (WPI3 and 4); Lanes 3 and 4: positive sera for *Babesia* sp. Tianzhu (WPI3 and 4); Lanes 5 and 6: positive sera for *Babesia* sp. BQ1 (Ningxian) (WPI3 and 4); Lanes 7 and 8: positive sera for *Babesia* sp. Hebei (WPI3 and 4); Lanes 9 and 10: positive sera for *Babesia* sp. Xinjiang (WPI3 and 4); Lanes 11-13: positive sera for *B. bigemina*, *B. bovis* and *T. luwenshuni*; and Lane 14: pre-immunized sheep (No. 3216) sera were used as the negative controls

The reactivity of each positive serum that was obtained from infected sheep with rRAP-1a61-1 and rRAP-1a61-1/CT was analyzed using Western blot analysis. Strong reactions showing an expected size of rRAP-1a61-1/CT (approximately 20 kDa) were observed in the positive sera obtained from sheep infected with *Babesia* sp. BQ1 (Lintan) (No. 3216), *Babesia* sp. BQ1 (Ningxian) (No. 08026) and *Babesia* sp. Tianzhu (No. 08040). In contrast, no reaction was observed between rRAP-1a61-1/CT and sera obtained from sheep infected with *Babesia* sp. Hebei (No. 08020) and *Babesia* sp. Xinjiang (No. 3201) (Fig. 3). Furthermore, cross-reactivity between the full-length rRAP-1a61-1 protein and sera from *Babesia* sp. Xinjiang-infected sheep and *B. bigemina*-infected cattle was also observed (data not shown).

### Identification of native RAP-1 proteins using Western blot analysis and IFAT

**Fig. 4:**
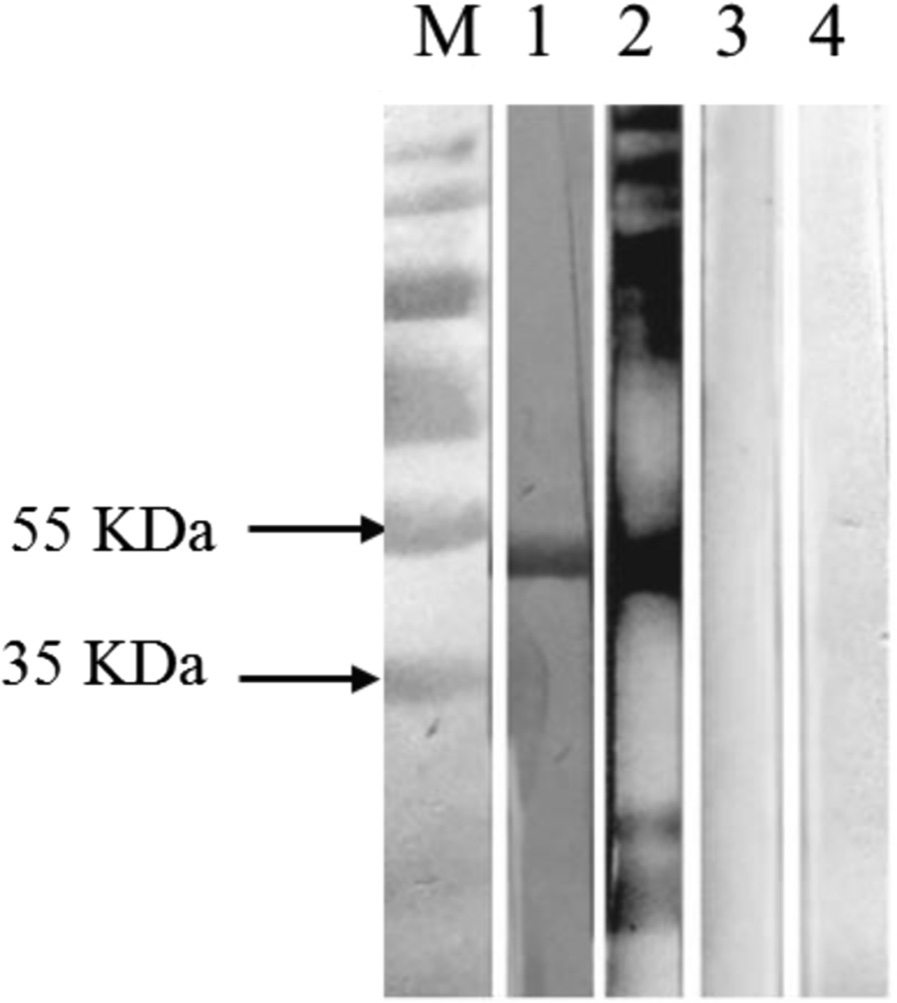
Western blot analysis of native RAP-1 in *Babesia* sp. BQ1 (Lintan) merozoite lysates. Lysates from *Babesia* sp. BQ1 (Lintan)-infected sheep erythrocytes were recognized by the serum of a rabbit that was immunized using rRAP-1a61-1/CT. Positive serum from *Babesia* sp. BQ1 (Lintan) (No. 3216) (Lanes 1 and 2). Pre-immunized sheep (No. 3216) and rabbit sera (Lanes 3 and 4) were used as the negative controls

To identify native RAP-1 in *Babesia* sp. BQ1 (Lintan) merozoites, antibodies against the C-terminal-truncated recombinant RAP-1a61-1/CT were obtained from rabbits and the positive serum obtained from animals infected with *Babesia* sp. BQ1 (Lintan) (No. 3216), and the RAP-1a61-1 protein was detected at approximately 52 kDa in the lysates of *Babesia* sp. BQ1 (Lintan)-infected sheep erythrocytes (Fig. 4). No specific reaction was observed in Western blot analyses between the sera with the lysates of uninfected sheep erythrocytes (data not shown).

**Fig. 5:**
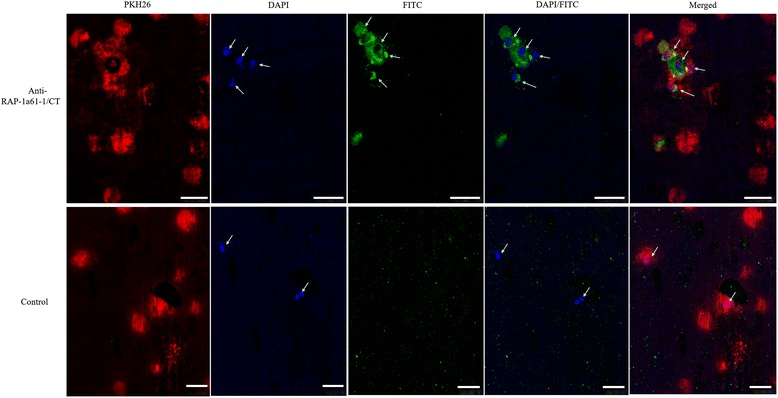
Identification of native RAP-1 proteins on *Babesia* sp. BQ1 (Lintan) parasites using immunofluorescence analysis. *Babesia* sp. BQ1 (Lintan)-infected red blood cells were stained with rabbit pre-immune serum (control) and anti-RAP-1a61-1/CT antibodies. RBCs were stained with PKH26 (shown in *red*). An anti-RAP-1a61-1 antibody (*green*) reacted with native RAP-1a on merozoites. Nuclei were counterstained with DAPI (*blue*). Merged images are shown to the right. Arrowheads indicate the merozoites. *Scale-bars*: 7.5 μm

The IFAT results showed that the merozoite nucleus of parasites were stained with DAPI and FITC-conjugated anti-rabbit IgG is able to detect anti-rRAP-1a61-1/CT antiserum. The immunofluorescence signals are the result of the interaction between the serum from a rabbit that was immunized by rRAP-1a61-1/CT and *Babesia* sp. BQ1 (Lintan) merozoites. Specific fluorescent signaling was not detected using pre-immune rabbit serum (Fig. 5).

### Cross-reactivity with other hemoparasites using ELISA

**Fig. 6:**
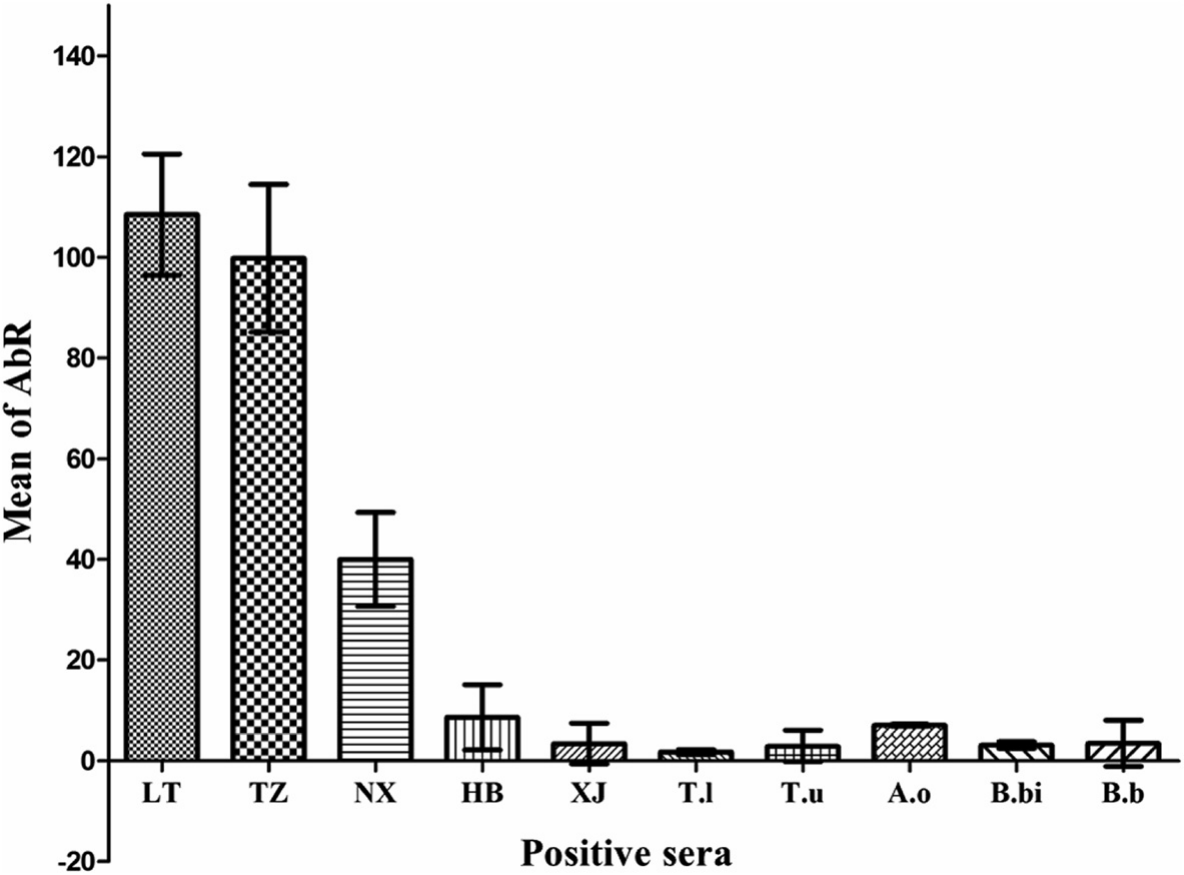
The cross-reactivity of rRAP-1a61-1/CT from *Babesia* sp. BQ1 (Lintan) with positive sera against *Babesia-*, *Theileria-* and *Anaplasma*-infected sheep or cattle was determined using ELISA. LT: *Babesia* sp. BQ1 (Lintan), TZ: *Babesia* sp. Tianzhu, NX: *Babesia* sp. BQ1 (Ningxian), HB: *Babesia* sp. Hebei, XJ: *Babesia* sp. Xinjiang, T.l: *T. luwenshuni*, T.u: *T. uilenbergi*, A.o: *A. ovis*, B.bi: *B. bigemina* and B. bo: *B. bovis*

Positive sera against *Babesia* sp. BQ1 (Lintan), *Babesia* sp. BQ1 (Ningxian), *Babesia* sp. Tianzhu, *Babesia* sp. Hebei, *Babesia* sp. Xinjiang, *T. lunwenshuni*, *T. uilenbergi*, *A. ovis*, *B. bovis*, and *B. bigemina* were tested using ELISA to evaluate cross-reactivity with rRAP-1a61-1/CT. The tests were repeated in triplicate, and the specific antibody mean rate (AbR % = (Sample mean OD-Negative control mean OD)/(Positive control mean OD-Negative control mean OD) × 100) and standard deviation were calculated using Excel 2007 [38]. The results indicated that cross-reactivity was observed only between three *B. motasi*-like isolates (Lintan, Tianzhu and Ningxian) and that no cross-reactivity was observed with the sera against *Babesia* sp. Hebei, *T. lunwenshuni*, *T. uilenbergi*, *B. bovis*, *B. bigemina* or *A. ovis* (Fig. 6).

### The kinetics of anti-*Babesia* sp. BQ1 (Lintan) antibodies were tested in experimentally infected sheep using ELISA

**Fig. 7:**
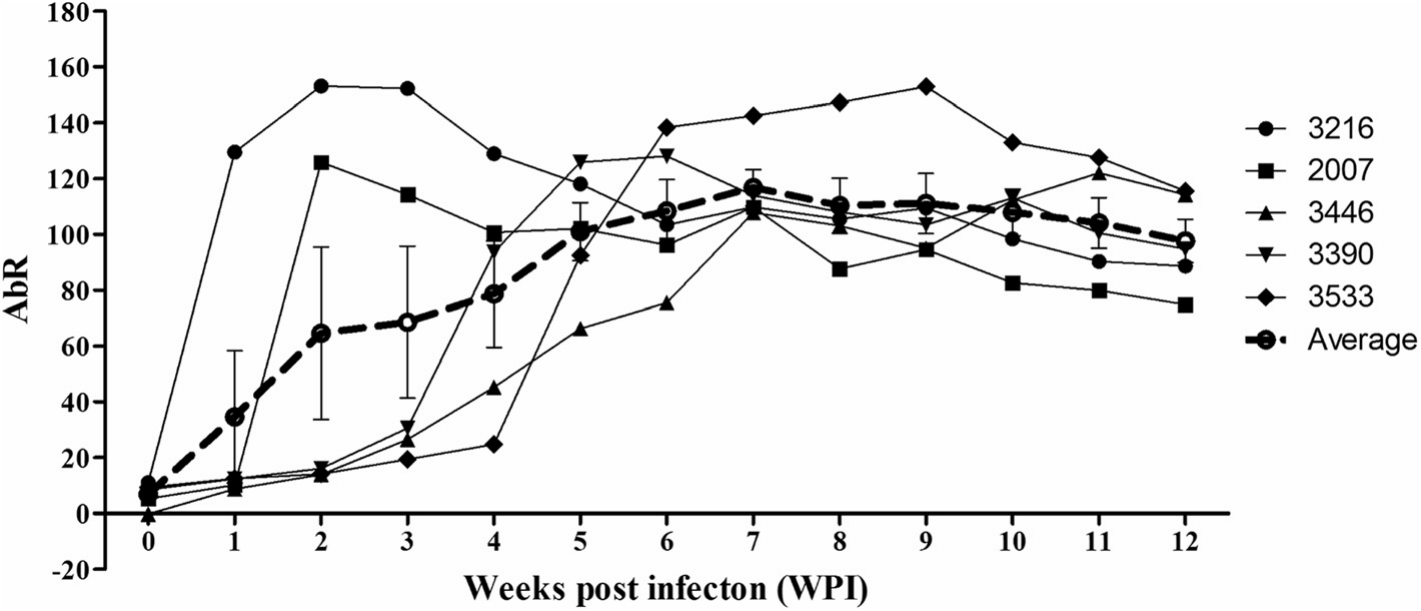
Kinetics of anti-*Babesia* sp. BQ1 (Lintan) antibodies in 5 sheep (Nos. 3216, 2007, 3446, 3390 and 3533) that were experimentally infected. The error bars indicate the standard deviations, and the bold dotted line shows the mean AbR that corresponded to each time point

The kinetics of antibodies produced against RAP-1a have been studied using ELISA with rRAP-1a61-1/CT in sera collected from 5 intact infected sheep (Nos. 3216, 2007, 3390, 3533 and 3446) before (0 weeks post-infection, WPI0) and after infection (from WPI1 to WPI12). A significant increase in the amount of antibodies produced against RAP-1a was observed after the sheep were infected, but varying kinetics were observed between individual sheep. In two of the Chinese sheep (Nos. 3216 and 2007), antibody production against RAP-1a was increased during the early phase of infection, at 1–3 weeks (WPI1-3), after which it decreased. In contrast, a gradual increase in the number of antibodies produced against RAP-1a was observed in the three French sheep (Nos. 3390, 3533 and 3446). Higher antibody levels were observed at 6–7 weeks post-infection (WPI6-7) in sheep Nos. 3390 and 3446 and at 9 weeks (WPI9) in sheep No. 3533. In general, antibodies were produced at 1 week post-inoculation, continued to increase through 6 weeks (WPI6), and remained stable until 9 weeks (WPI9), after which slight decreases were observed through 12 weeks (WPI9-12). High levels of antibodies against RAP-1a usually lasted 3 months (84 days p.i.), despite the decrease in production that was observed after 6 weeks (42 days p.i.) (Fig. 7).

### Evaluation of specificity, sensitivity and positive threshold values in ELISA to detect rRAP-1/CT

**Fig. 8:**
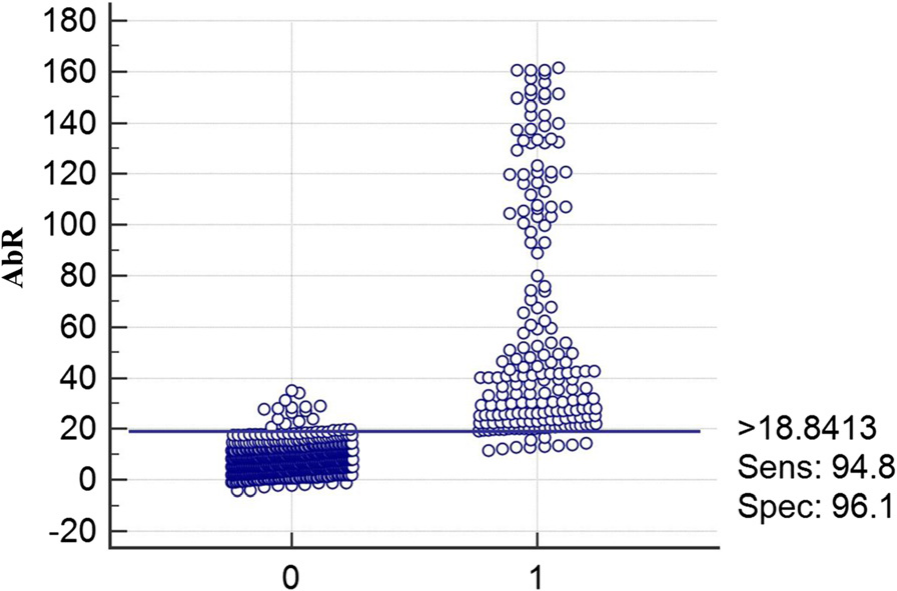
Evaluation of the specificity, sensitivity and positive threshold value of rRAP-1/CT in ELISA using antibody mean rates (AbRs) for positive and negative sera. 0 = negative sera, and 1 = positive sera

MedCalc statistical software was used to evaluate the sensitivity, specificity, and positive threshold value in ELISAs performed to test 191 positive sera and 492 negative sera, as previously described [40]. A percentage corresponding to the specific antibody mean rate (AbR %) was calculated for each serum sample. The results indicated that the threshold value was 18.84 %, which corresponded to 94.8 % sensitivity (95 % CI = 82.4–98.4) and 96.1 % specificity (95 % CI = 85.1–99.4). The numbers of false positive and false negative sera were 10 and 15, respectively (Fig. 8).

### ELISA identification of the rRAP-1a61-1/CT protein as a potential antigen for use in serological epidemiology in *B. motasi*-like sub-group (Lintan, Ningxian and Tianzhu isolates) infections

Field serum samples were extracted from a total of 3198 sheep and goats from 54 different regions of 23 Chinese provinces. The samples were analyzed using ELISA to evaluate the prevalence of infection with *B. motasi*-like (Lintan, Ningxian and Tianzhu) isolates in these regions. The results of ELISA detection to identify positive samples are summarized in Table 1. Sero-positive with *B. motasi*-like were identified in 51 regions covering all 23 surveyed provinces. The total positive rate in these regions was 36.02 % (95 % CI = 33.7–68.4). The sero-prevalence ranged from 2.22 to 81.11 %. The significantly high positive rates exceeded 50 % in 7 of the 23 surveyed provinces, including Liaoning (50 %), Inner Mongolia (61.01 %), Shaanxi (75.68 %), Hunan (52.73 %), Sichuan (59.72 %), Anhui (81.11 %) and Shanxi (58 %). Three additional provinces, including Qinghai (45.35 %), Ningxia (49.38 %) and Guangdong (41.89 %), had positive rates exceeding 40 %. Lower sero-prevalence was observed in Tibet (2.22 %), Jilin (7.55 %) and Guangxi provinces (9.74 %). Positive rates that were greater than 80 % were observed in Chifeng (86.15 %) in Inner Mongolia and Lintan (83.33 %) in the Gansu provinces (Table 1, Fig. 1).

**Table 1:** Prevalence of *Babesia* sp. BQ1 Lintan in field samples collected from 23 provinces by detected antibodies produced from rRAP-1a CT with ELISA

Province	Prefecture	No. of sera	No. of positive sera	Positive rate (%) in different regions	Positive rate (%) in each province
Qinghai	Haibei	269	122		(122/269) 45.35
Xinjiang	Yili	229	82	35.81	(118/419) 28.16
	Akesu	90	20	22.22	
	Habahe	100	16	16	
Gansu	Jingtai	120	8	6.67	(160/447) 35.79
	Jiayuguan	81	6	7.41	
	Tianzhu	81	56	69.14	
	Yongchang	75	15	20	
	Lintan	90	75	83.33	
Tibet	Lhasa	90	2		(2/90) 2.22
Liaoning	Huanren	29	14	48.28	(42/84) 50
	Anshan	27	10	37.04	
	Fengcheng	28	18	64.29	
Jilin	Yongji	26	3	11.54	(4/53) 7.55
	Jiutai	27	1	3.70	
Inner Mongolia	Ordos	63	21	33.33	(133/218) 61.01
	Manzhouli	13	0	0	
	Baotou	12	0	0	
	Chifeng	130	112	86.15	
Shaanxi	Yulin	74	56		(56/74) 75.68
Ningxia	Wuzhong	81	40		(40/81) 49.38
Guangxi	Jingxi	35	3	8.57	(15/154) 9.74
	Tianyang	56	10	17.85	
	Pingxiang	18	0	0	
	Guilin	45	2	4.44	
Hunan	Xinhuang	29	16	55.17	(29/55) 52.73
	Xiangtan	26	13	50	
Sichuan	Panzhihua	36	25	69.44	(43/72) 59.72
	Hejiang	36	18	50	
Yunnan	Fuyuan	35	2	5.71	(65/202) 32.18
	Honghe	32	16	50	
	Jinghong	20	5	25	
	Yanshan	34	10	29.41	
	Menghai	12	3	25	
	Ruili	32	14	43.75	
	Kunming	37	15	40.54	
Anhui	Hefei	90	73		(73/90) 81.11
Guangdong	Zhaoqing	38	20	52.63	(31/74) 41.89
	Lianzhou	36	11	30.56	
Guizhou	Dushan	30	1	3.33	(46/166) 27.71
	Ziyun	32	13	40.63	
	Yuping	32	19	59.38	
	Guiyang	72	13	18.06	
Chongqing	Jiangjin	30	11	36.67	(15/53) 28.30
	Wanzhou	23	4	17.39	
Shandong	Dongying	90	19		(19/90) 21.11
Shanxi	Lvliang	50	29		(29/50) 58
Zhejiang	Jingning	36	15	41.67	(32/117) 27.35
	Qingtian	29	4	13.79	
	Hangzhou	52	13	25	
Hubei	Suizhou	81	27		(27/81) 33.33
Hebei	Baoding	169	25		(25/169) 14.79
Henan	Linzhou	60	20	33.33	(26/90) 28.89
	Neihuang	30	6	20	
Total		3198	1152		(1152/3198) 36.02

## Discussion

In the genus *Babesia*, RAP-1 was initially described according to its recognition by monoclonal antibodies to merozoite antigens in *B. bigemina.* It was designated p58 at that time [41], and it was subsequently identified in all *Babesia* species that were examined. The sequence of the full-length *rap-1* gene was first published in *B. bovis* (named pBv60) [18], and the sequence and multigene nature of p58 (later named *rap-1a*) were described in *B. bigemina* [42]. The name RAP-1 was first applied when *B. bovis* pBv60 was found to be localized in rhoptries under immunoelectron microscopy [27]. The definitive structure of the *rap-1* locus was initially defined in *B. bovis* [43] and then in *B. bigemina* [44]. In summary, an antibody reacting with a *B. bigemina* protein (p58) that was later identified as *rap-1* was reported before its *B. bovis* homologue, and the sequence features, the association of these proteins with rhoptries, and the detailed organization of the locus containing *rap-1* were initially described in *B. bovis*. In all studied *Babesia* species, the RAP-1 proteins share common conserved features that include the strict conservation of 4 cysteine residues, a highly conserved 14 amino acid motif and several shorter conserved oligopeptide motifs in the first N-terminal 300 amino acids. Among babesial parasites, the N-terminal regions of RAP-1 are more conserved than those in the C-terminal region [32, 35].

In bovine *Babesia* species, previous studies have indicated that the full-length recombinant RAP-1a in *B. bovis* could be used as a diagnostic antigen in ELISA to detect antibodies and to show cross- reactivity with *B. bigemina* because of the conserved features in the N-terminus of RAP-1, as described above [29]. In contrast, RAP-1 B-cell epitopes in *B. bovis* and *B. bigemina* have been mapped using monoclonal antibodies and immune sera obtained from cattle, and the results demonstrated that the B-cell epitopes primarily targeted the C-terminal region of the proteins [27, 45, 46]. Moreover, an ELISA based on recombinant RAP-1a that is modified in the C-terminal region has been developed to improve its sensitivity and specificity for antibody detection and could be widely used as an antigen for serodiagnosis and epidemiological survey purposes [37, 47–50]. In *B. motasi*-like isolates, software prediction of RAP-1a B-cell epitopes demonstrated that B-cell epitopes were also located in the C-terminal region. Only a few B cell-epitopes were predicted in the N-terminal region (data not shown). Interestingly, the amino acid sequences between RAP-1a61 and RAP-1a67 are more conserved at the C-terminal region than the N-terminal region in *B. motasi*-like [34], which is not consistent with the common features described above. Moreover, the sequences of RAP-1a and the full *rap-1* locus, including the intergenic regions in three isolates (Lintan, Ningxian and Tianzhu) of *B. motasi*-like, were almost identical [12]. The features of the more conserved C-terminal region of the RAP-1a protein in *B. motasi*-like could indicate that this form is an ideal candidate for diagnostics aimed at identifying several *B. motasi*-like isolates.

The sera were able to recognize the native RAP-1 protein in Western blot analysis and IFAT, suggesting that the sheep had been immunized against RAP-1 during infection. Significant cross-reactivity was observed between rRAP-1a61-1/CT of *Babesia* sp. BQ1 (Lintan) and the positive sera obtained from *Babesia* sp. Tianzhu and *Babesia* sp. BQ1 (Ningxian) in both Western blot analyses and ELISA, but not in the Hebei isolate. This is not surprising because the *rap-1a* sequences were almost identical in these three *Babesia* isolates at both the nucleotide and amino acid level. In contrast, the sequences of all of the *rap-1* genes obtained from *Babesia* sp. Hebei were clearly different from the other 3 isolates, suggesting that this isolate might belong to a different species [12]. These findings were inconsistent with a previous study that indicated that weak cross-reactivity between *Babesia* sp. BQ1 (Ningxian) and *Babesia* sp. BQ1 (Lintan) was detected using Western blot analysis but not ELISA using a merozoite antigen (BQMA) [40]. Some novel antigens (e.g., BQP35 and BQHsp90) for *Babesia* sp. BQ1 (Lintan) were recently identified as candidate diagnostic antigens. Western blot analysis and ELISA tests that are based on BQP35 have shown that there is cross-reactivity between *Babesia* sp. BQ1 (Lintan) and *Babesia* sp. Tianzhu, which is in agreement with the results obtained when merozoite extracts were used [51]. However, rBQHSP90 could not differentiate between any *B. motasi*-like isolates and *Babesia* sp. Xinjiang, another distinct ovine *Babesia* species [52]. Differences in cross-reactivity have been observed when using the ELISA method based on BQP35, BQHsp90 or RAP-1a of *Babesia* sp. BQ1 (Lintan), and these might be caused by differences in molecular characterizations and the detection limitations and sensitivities of these antigens. These findings suggest that rRAP-1a61-1/CT could be used as a potentially diagnostic antigen to increase the sensitivity and specificity of such tests, and that it might replace native crude parasite antigens, which require parasite reproduction (from the killing of experimentally infected animals) or the in vitro harvesting of cultures [53, 54].

The kinetics analysis revealed that the humoral immune response to this parasite varied in different sheep breeds (Chinese and French sheep) during the course of infection. This result was inconsistent with a previous study that used BQMA and indicated that there is no difference in kinetics between these two sheep breeds [38]. Antibody production against RAP-1 was initially increased in the two infected Chinese sheep between WPI1 and WPI2, but it then decreased. These results were consistent with the findings of a recent study, in which a RAP-1a peptide designed from the RAP-1a N-terminal of *Babesia* sp. BQ1 (Lintan) was used as an antigen to perform ELISA. Antibody production against this peptide displayed kinetics that were similar to those observed against rRAP-1a61-1 (data not shown). In contrast, the production of specific antibodies against *Babesia* sp. BQ1 (Lintan) in the three infected French sheep was increased at three weeks post-infection and peaked between six and nine weeks post-infection. On average, RAP-1a was expressed at sequentially higher levels from week 1 to week 7 post-infection before its levels subsequently decreased. The difference in the kinetics of antibody production between these five infected sheep might result from differences in sheep breeds or individual variation. In general, our results are similar to those described in a previous study that used a merozoite antigen (BQMA) to evaluate the kinetics of antibody production during the course of *Babesia* sp. BQ1 (Lintan) infection. That study showed that antibody production was decreased from WPI 12 (approximately 80 DPI) [38].

A serological diagnostic test (ELISA) is the most practical and economical assay for a wide range of epidemiological investigative purposes, and it has been established as a method for evaluating the lysates of *Babesia*-infected erythrocytes in a number of *Babesia* species [55]. However, studies focusing on the development of a serological diagnostic ELISA test that is based on recombinant immune antigens that can detect ovine babesiosis are rare. The development of ELISA tests requires preliminary knowledge of parasite polymorphism, the ability to successfully culture cells in vitro to produce antigens, and/or prior genetic knowledge of the targeted antigen so that specific sequences can be selected as an epitope. Some improved ELISA methods for sero-epidemiological surveys have been developed based on specific recombinant proteins (RAP-1 and SBPs) that are involved in the RBC invasion process in *B. bovis*, *B. bigemina* and *B. caballi* species [36, 37, 50, 56]. In this study, the results of sero-investigations using an ELISA method that was developed based on the rRAP-1a61-1/CT protein indicated that *Babesia* sp. BQ1 (Lintan), *Babesia* sp. BQ1 (Ningxian) and *Babesia* sp. Tianzhu infections were identified in all of the investigated provinces. Recent large-scale sero-epidemiological ELISA surveys of *Babesia* sp. BQ1 (Lintan) were performed in small ruminants from 22 provinces of China, and these were also regions that were tested in our study, and the results indicated that infection by this isolate was identified in all 22 investigated provinces, with an average prevalence of 43.5 %. These values are slightly higher than the values produced in our study and they showed a positive rate that was as high as 91 % in Inner Mongolia [57]. The positive rates from 8 provinces that were tested using rRAP-1a were slightly higher**,** whereas the positive rates from 14 of the provinces were significantly lower in our study than in a previous study [57]. This variation may result from differences in the limitations of the tested antigens. Moreover, another sero-epidemiological investigation performed using ELISA to detect infections in small ruminants by *Babesia* species indicated that there was an average infection rate of 82.38 % for *Babesia* sp. BQ1 (Lintan) in 261 sera samples obtained from Lintan County in Gansu province, and these result were similar to our results (83.33 %) for this region [38].

The positive rates from 10 of the 23 provinces were comparable to those found in a recent molecular detection study that used a nested PCR assay based on the *rap-1b* gene of *B. motasi*-like species, the positive rates for *Babesia* sp. BQ1 (Lintan), *Babesia* sp. BQ1 (Ningxian) and *Babesia* sp. Tianzhu were 5, 13.8 and 21.9 % in Inner Mongolia, Gansu and Xinjiang, respectively in this previous study (they were 61.01, 35.79 and 28.16 %, respectively, in our study) [58]. No *B. motasi*-like infections were detected in DNA samples obtained from the Zhejiang, Guizhou, Chongqing and Liaoning provinces when the nested PCR test was used [58]. Larger variation was observed in the numbers of positive samples that were identified in these provinces when ELISA was used in our study. A similar study was performed that compared the results of using ELISA (66.84 %) and the molecular LAMP method (14.3 %) to analyze *Babesia* sp. BQ1 (Lintan) infections in Gansu province [38]. Therefore, the difference in prevalence that is observed when using different testing methods (e.g., PCR vs ELISA or LAMP vs ELISA) or different antigens (e.g., BQMA vs rRAP-1a) or when using the same method (e.g., ELISA) can be potentially explained by the parameters that were used or the limitations of the detection methods used in these assays.

Some studies have indicated that *Haemaphysalis qinghaiensis* and *H. longicornis* are the vectors of the *Babesia* sp. BQ1 (Lintan) isolate, while *H. longicornis* has been proposed as the transmission vector for *Babesia* sp. BQ1 (Ningxian). These two tick species are widely distributed in at least 20 provinces of China (Fig. 1) [4, 59, 60]. Whereas, the higher positive rates (exceed 25 %) of *B. motasi*-like were found in Inner Mongolia, Xinjiang, Hunan, Zhejiang, Guangdong and Chongqing, as well as in Guangxi with positive rate of 9.74 % where the *H. longicornis* and *H. qinghaiensis* have not been reported yet. This has been explained by several possibilities by Wang et al. [57]. Moreover, cases of ovine babesiosis have been reported in many Chinese provinces, including Inner Mongolia, Henan, Shaanxi, Yunnan, Sichuan, Heilongjiang and Qinghai province [61–63]. Taken together, the results show that the wide prevalence of the *B. motasi*-like isolates (Lintan, Ningxian and Tianzhu) included in this study and co-infections between these three isolates is not surprising, and these isolates co-circulate in China with high prevalence. The results suggest that the development of ELISA methods that are based on the rRAP-1a61-1/CT described in our study indicate that this ELISA is a potential diagnostic method for epidemiological investigations of known related isolates in addition to other unidentified isolates in sheep *B. motasi*-like infections.

## Conclusions

The present study demonstrates that antibodies against rRAP-1a61-1 can detect native RAP-1 in *Babesia* sp. BQ1 (Lintan) merozoite lysates in Western blot analysis and IFAT. The high conservation of RAP-1a across the three isolates from *B. motasi*-like parasites suggests that rRAP-1a61-1/CT could be a promising common diagnostic antigen that can detect geographically distinct isolates of *B. motasi*-like infections in sheep.
